# Plant-Based Dietary Patterns and the Risk of Cardiovascular Disease in Middle-Aged Korean Adults: A Community-Based Prospective Cohort Study

**DOI:** 10.3390/nu17172805

**Published:** 2025-08-28

**Authors:** Chaeyoung Park, Boeun Han, Yujin Lee

**Affiliations:** Department of Food and Nutrition, Myongji University, Yongin 17058, Republic of Korea

**Keywords:** cardiovascular disease, plant-based diets, unhealthful plant-based diet, healthful plant-based diet

## Abstract

**Background/Objective**: Plant-based diets are gaining global attention for their positive impact on health and sustainability; however, the nutritional value and health effects differ across plant food categories. We investigated the association of three plant-based diet indices and incident cardiovascular disease (CVD) and its subtypes. **Methods:** This study consisted of 10,030 Korean adults aged 40–69 years from the Korean Genome and Epidemiology Study (KoGES) in Ansan and Ansung. Using a validated food frequency questionnaire from the community-based cohorts of the KoGES, we derived three dietary indices based on food intake: (1) Overall Plant-Based Diet Index (PDI), (2) Healthful Plant-Based Diet Index (hPDI), and (3) Unhealthful Plant-Based Diet Index (uPDI). We analyzed the association between three plant-based diet indices and the incidence of CVD using a multivariate Cox proportional hazards regression model, adjusted for demographic and other CVD risk factors. **Results:** During 99,751 person-years, 597 CVD cases occurred. None of the three plant-based diet indices (PDI, hPDI, uPDI) were significantly associated with overall risk of CVD. When stratifying results by types of CVD, individuals with the highest adherence to uPDI had a higher risk of coronary heart disease (CHD), compared to the lowest group [HR (95% CI) = 1.62 (1.12–2.33), *p*-trend = 0.008], but not stroke [HR (95% CI) = 0.97 (0.66–1.42), *p*-trend = 0.964]. There were no associations between adherence to PDI and hPDI and the incidence of CHD and stroke. **Conclusions:** In this prospective cohort of Korean adults, none of the three plant-based diet indices were associated with CVD risk, whereas higher adherence to an unhealthful plant-based diet was associated with increased risk of CHD, but not stroke. These findings highlight the importance of plant food quality in CHD prevention and warrant confirmation in other populations.

## 1. Introduction

Cardiovascular disease (CVD) is the foremost contributor to global morbidity and mortality, responsible for approximately 19.8 million deaths each year, with its impact increasingly evident in low- and middle-income countries [1]. In Korea, CVD is also a major public health concern, with its prevalence expected to rise as the population ages and adopts more Westernized dietary patterns [2]. Diet is a modifiable risk factor for CVD, and increasing attention has been directed toward plant-based diets as a strategy for prevention [3].

Plant-based dietary patterns have become increasingly prevalent, driven by both their potential health-promoting properties and their relevance to environmental sustainability [4]. By delivering essential vitamins, minerals, and antioxidant-rich fiber, these diets help mitigate oxidative and inflammatory processes, enhance insulin action, and maintain healthy vascular and glycemic status [5,6]. Together, these processes could contribute to reducing CVD risk. A growing body of evidence suggests that higher intake of plant foods is linked to a reduced risk of non-communicable diseases [7,8]. However, not all plant-based foods are equally healthful. Diets high in refined grains, sugar-sweetened beverages, and salty snacks—though plant-derived—may contribute to adverse health outcomes [9,10,11]. This has led researchers to distinguish between healthful and unhealthful plant-based diets, developing three plant-based diet indices [12]: the overall Plant-Based Diet Index (PDI), which emphasizes plant foods regardless of their health quality; the healthful PDI (hPDI), which assigns higher scores to nutrient-dense plant foods such as fruits, vegetables, and whole grains; and the unhealthful PDI (uPDI), which gives higher scores to less nutritious plant-based foods, including refined carbohydrates and sugary items. These indices allow for a more comprehensive evaluation of plant-based dietary patterns and their associations with health outcomes.

Although prior studies have demonstrated inverse associations of healthful plant-based dietary patterns with CVD and related risk factors, most research has been conducted in Western populations [12,13,14,15,16]. Evidence from Asian populations, particularly those with distinct dietary patterns and chronic disease profiles, remains limited. In addition, the differential associations of plant-based diet quality with specific CVD subtypes, such as coronary heart disease (CHD) and stroke, remain unclear.

To address these gaps in knowledge, we aimed to investigate the associations of overall, healthful, and unhealthful plant-based diet indices (PDI, hPDI, and uPDI) with incident CVD and its subtypes in a large, prospective cohort of Korean adults. By examining both total CVD and its major components, this study provides novel insights into the potential differential impact of plant-based diet quality on distinct cardiovascular outcomes.

## 2. Materials and Methods

### 2.1. Study Design and Population

**Figure 1:**
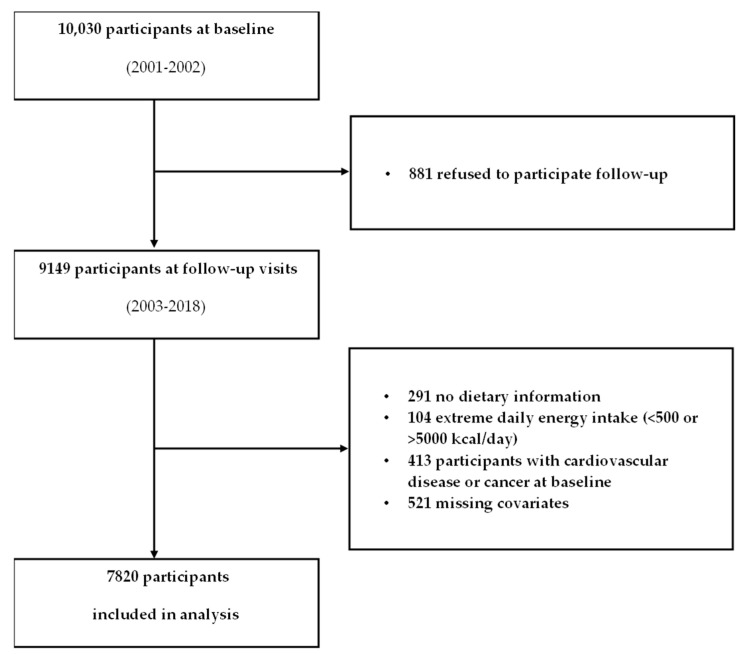
Flowchart of the study.

The Korean Genome and Epidemiology Study (KoGES)_Ansan and Ausung study is a population-based cohort initiated in 2001 by the Korean Disease Control and Prevention Agency, following residents of an urban city (Ansan) and a rural community (Ansung) to investigate determinants of chronic illnesses in adults aged 40 years and older [17]. A total of 10,030 individuals aged 40–69 years, including 5012 men and women in Ansan and 5018 men and women in Ansung, were recruited in 2001–2002. Since the baseline survey of 10,030 individuals in 2001–2002, follow-up surveys have been conducted every two years. In this study, data from the baseline survey in 2001–2002 to the 8th follow-up survey in 2017–2018 were utilized for analysis. Trained research staff, who received centralized training in the standardized KoGES protocol developed by the Korea Disease Control and Prevention Agency, conducted interviews to assess participants’ demographic characteristics, lifestyle, and medical history. These personnel were specifically trained to administer questionnaires (including the FFQ, medical history, and lifestyle factors) and to perform anthropometric measurements following uniform procedures to ensure data consistency and reliability [17]. Among the 10,030 individuals surveyed at baseline, those who met any of the following criteria were excluded from the study: individuals with any missing responses in the food frequency questionnaire (FFQ) data (*n* = 326), individuals with extreme energy intake levels (<500 kcal/day or >5000 kcal/day) (*n* = 110; based on pre-specified plausibility cutoffs and not related to the residual method), individuals diagnosed with CVD or cancer at baseline (*n* = 453), individuals who did not participate in follow-up surveys after the baseline survey (*n* = 800), and individuals with missing information on covariates including age, sex, residence area, income, education level, smoking, alcohol consumption, physical activity, BMI, total energy intake, family history of CVD, and history of hypertension (*n* = 521). Medication use was not an exclusion criterion, and potential confounding by underlying conditions was accounted for by multivariable adjustment. As a result of these exclusions, a total of 7820 individuals (3721 men and 4099 women) were included in the analysis (Figure 1). The KoGES obtained research approval from the Institutional Review Board (IRB) of the Korea Centers for Disease Control and Prevention, and all participants provided written informed consent. This study was conducted with approval from the IRB of Myongji University (No. MJU-2022-08-002-01). This cohort analysis was prepared in accordance with the STROBE (Strengthening the Reporting of Observational Studies in Epidemiology) guidelines for cohort research (Table S1) [18].

### 2.2. Calculation of Plant-Based Diet Index Scores

We collected dietary information at two time points (2001–2002 and 2005–2006) with a semi-quantitative FFQ that included nine frequency options—from “never” to “three times a day”—to capture usual intake over the prior 12 months [19]. Nutrient intake was calculated by multiplying the reported consumption frequency and portion size of each food item by its nutrient composition values obtained from the Food Composition Table published by the Korean Nutrition Society. The daily nutrient intake of each participant was determined by summing these values across all food items [20]. To capture long-term dietary habits, cumulative averages of dietary intake were used. Specifically, dietary intake reported in 2001–2002 was related to CVD events occurring between 2001–2002 and 2005–2006, while dietary data from 2001–2002 and 2005–2006 were averaged and applied to events recorded between 2005–2006 and 2018. For participants with missing dietary data in 2005–2006, baseline dietary intake from 2001–2002 was carried forward.

Three indices reflecting plant-based eating patterns (PDI, hPDI, and uPDI) were created from FFQ data. The 106 reported items were grouped into 17 categories based on nutrient profile and culinary similarity, then sorted into three broader classes: healthful plant foods, less-healthful plant foods, and animal-derived foods. This classification was guided by previous evidence linking food types to chronic disease and metabolic outcomes [21,22]. Healthy plant foods encompassed items like whole grains, vegetables, fruits, legumes, nuts, tea, and coffee. Refined grains, potatoes, sugar-sweetened beverages, desserts, pickled vegetables, and sauces were labeled less-healthy, and foods such as dairy, eggs, meats, fish, and animal fats were classified as animal foods (Table S2). Intake of each food group was adjusted for total energy intake using the residual method when deriving the plant-based diet indices (PDI, hPDI, uPDI) [23,24]. This energy adjustment was performed exclusively for diet index calculation and was not related to the exclusion of participants with extreme energy intake. Each of the 17 food groups was ranked into five categories according to consumption, with scores assigned from 1 to 5. For the PDI, participants in the highest quintile of plant food consumption received a score of 5 for each plant-based food group, while those in the lowest quintile received a score of 1; scores for animal food groups were assigned in reverse. For the hPDI, positive scores were given to healthy plant food groups, while less healthy plant food groups and animal food groups were scored in reverse. In the uPDI, positive scores were assigned to less healthy plant food groups, with reverse scoring applied to healthy plant food groups and animal food groups. Scores from all food groups were added to form a total index, which was grouped into four categories (quartiles) for evaluation. The possible values for PDI, hPDI, and uPDI spanned 17 to 85.

### 2.3. Ascertainment of Cardiovascular Disease

CVD was defined as encompassing myocardial infarction, coronary artery disease, stroke, congestive heart failure, and peripheral vascular disease. Incident CVD history for each participant was collected via self-reported questionnaires administered by trained staff at follow-up visits. In this study, CVD subtypes refer to CHD (including myocardial infarction and coronary artery disease) and stroke; however, information on stroke subtypes (ischemic vs. hemorrhagic) was not available from the questionnaire. If a participant reported a new CVD event, in-depth personal interviews were conducted and a review of their clinical documentations, such as death certificate, autopsy reports, medical records, hospital discharge summaries, and laboratory results, was carried out to confirm the case [25].

### 2.4. Covariates

At each biennial follow-up, participants underwent standardized clinical evaluations consisting of structured interviews and physical examinations. The full protocol has been described previously [17]. Briefly, trained personnel collected information on sociodemographic characteristics, lifestyle factors (including diet, smoking, alcohol consumption, and physical activity), and both personal and family medical histories. Anthropometric measurements were obtained using standardized procedures. Residential location was classified as urban (Ansan) or rural (Ansung), educational attainment was categorized as ≤elementary school, middle school, high school, or ≥college, and monthly household income was grouped into four levels: <1,000,000 KRW, 1,000,000–<2,000,000 KRW, 2,000,000–<3,000,000 KRW, and ≥3,000,000 KRW. Smoking was assessed in pack-years, calculated by multiplying the number of packs smoked per day by the number of years smoked. Alcohol consumption was assessed using a questionnaire that first classified participants as lifetime abstainers, former drinkers, or current drinkers. Only participants who reported current drinking were asked to provide the amount of alcohol consumed, and their responses were converted into a continuous variable representing total alcohol intake (g/day). Physical activity was quantified by assigning metabolic equivalent task (MET) values to activity intensities and calculating the corresponding activity time, thereby representing the duration of physical activity during daily tasks [26,27]. Family history of myocardial infarction, congestive heart failure, coronary artery disease, peripheral vascular disease or cerebrovascular disease was categorized as “yes” if participants responded with “father”, “mother”, “siblings”, or “other”, and as “no” if there was no family history. Anthropometric data were obtained while subjects wore minimal clothing and no footwear, and values were recorded to 0.1 cm for height and 0.1 kg for weight.

### 2.5. Statistical Analysis

To assess associations between diet indices and CVD risk, we fitted Cox proportional hazards models (stcox command in Stata/SE version 17.0). Events were defined as any incident CVD during the study period; censoring occurred at the earliest date of non-CVD death, dropout, or the last observation in 2018. The Cox proportional hazards assumption was evaluated using a test based on Schoenfeld residuals [28]. To mitigate residual confounding, analyses were adjusted for a predefined set of covariates identified on the basis of biological plausibility, established cardiovascular risk factors, and their potential associations with plant-based diet indices and CVD outcomes. The variables included age, sex, residential location, household income, educational level, smoking status, alcohol consumption, family history of cardiovascular disease (myocardial infarction, heart failure, coronary artery disease, peripheral vascular disease, and cerebrovascular disease), history of hypertension, BMI, physical activity, and total energy intake. Potential nonlinear associations of each plant-based diet index with risk of CVD were explored using restricted cubic splines with three knots. To compare the nutrient density according to adherence to dietary patterns, the macronutrients (carbohydrates, protein, and fat) were expressed as the proportion of total energy intake, and vitamins and minerals were analyzed based on intake per 1000 kcal. We evaluated potential effect modification in the associations between plant-based diet quality and CVD by age (40–49, 50–64, ≥65 years), sex, family history of CVD (yes/no), physical activity (median METs/day), BMI (<18.5, 18.5–22.9, 23.0–24.9, ≥25.0 kg/m^2^), smoking (packs/year), and history of hypertension (yes/no). Multiplicative interaction terms were tested using Wald statistics, and no significant interactions were detected (*p* for interaction ≥ 0.007, Bonferroni-adjusted threshold = 0.05/7). Linear trend tests were conducted by assigning the median score of each quartile to participants and modeling it as a continuous variable. For the exploratory analysis, CVD was further disaggregated into CHD and stroke. Additionally, potential effect modification of the relationship between each plant-based diet index and CHD or stroke was explored for age, sex, BMI, family history of CHD or stroke, physical activity, smoking, and history of hypertension. Statistical significance of each multiplicative interaction term was assessed using the Wald test, with *p* values for these exploratory analyses. Bonferroni adjusted for multiple comparisons (α = 0.05/7 = 0.007). Sensitivity analyses were conducted excluding early events (within the first 2 years) to minimize any effect of preexisting subclinical disease leading to change in adherence to plant-based diet patterns to minimize reverse causation. All statistical analyses were performed with STATA/SE version 17.0 (StataCorp LLC, College Station, TX, USA), and results with *p* values below 0.05 were considered significant.

## 3. Results

### 3.1. Baseline Characteristics

At baseline, participants had a mean (SD) age of 52.0 (8.8) years, 52.4% were female, and 51.8% resided in the urban site (Ansan). PDI scores ranged from 31 to 70, hPDI from 29 to 74, and uPDI from 30 to 75. In unadjusted analyses, those in the higher PDI and hPDI categories were more often women, older, rural residents, less educated, had lower income, fewer smoking pack-years, consumed less alcohol, had higher BMI and physical activity, a greater prevalence of hypertension, and reported higher intake of plant foods and lower intake of animal foods compared with those in the lower categories. Higher PDI was associated with lower total energy intake, whereas higher hPDI corresponded to greater energy intake. Conversely, individuals with higher uPDI were more often men, older, rural, less educated, with lower income, heavier smoking histories, higher alcohol consumption, lower BMI, and greater physical activity. They also exhibited a higher prevalence of hypertension, higher energy intake, and greater consumption of less healthful plant foods, with reduced intake of both healthful plant foods and animal foods (all *p* < 0.001). No differences were observed across any diet score in the prevalence of family history of CVD (Table 1).

### 3.2. Nutrient Intakes Across Quartiles of Plant-Based Diet Indices

Higher adherence to the overall PDI was linked to greater energy from carbohydrates and increased intake of numerous vitamins and minerals, such as vitamins A, E, C, B6, folate, niacin, calcium, phosphorus, sodium, potassium, iron, and fiber, with lower contributions from protein, fat, vitamin B2, zinc, and cholesterol. Elevated hPDI scores reflected similar patterns but with reduced levels of some nutrients (e.g., vitamin A, B2, niacin, calcium, sodium, zinc). In contrast, higher uPDI scores were characterized by more carbohydrate and sodium but generally lower levels of other micronutrients (Table 2).

### 3.3. Association of Plant-Based Diet Indices with CVD

**Figure 2:**
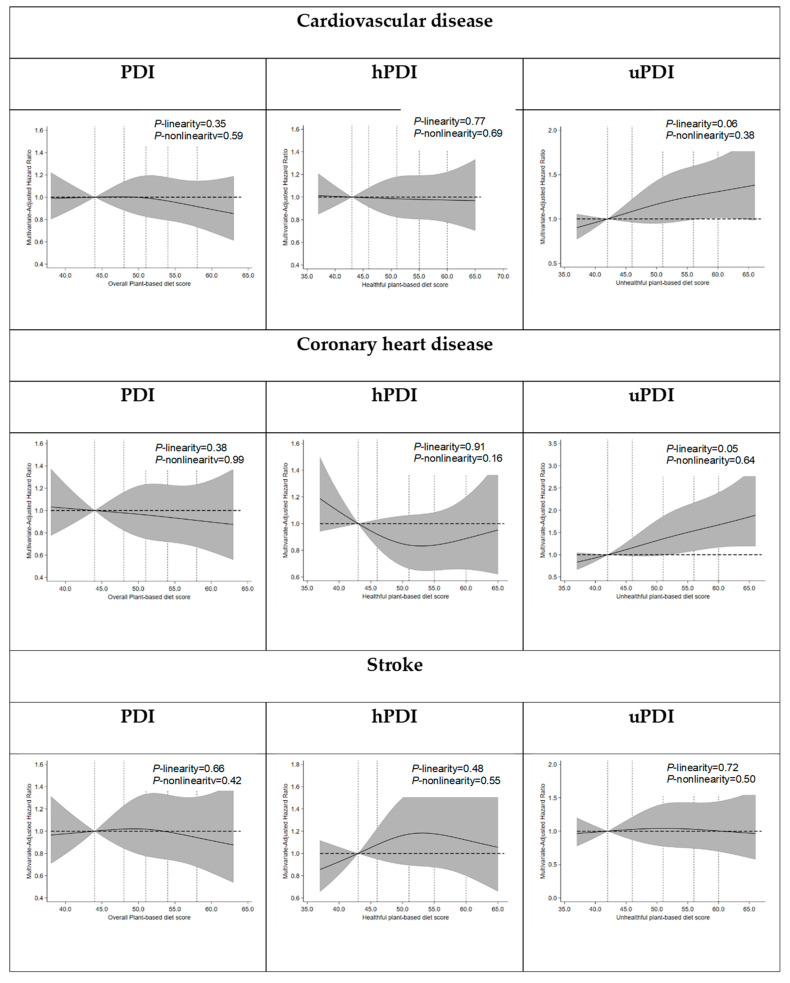
Multivariable-adjusted relationship of plant-based diet indices with risk of cardiovascular disease and its major subtypes: coronary heart disease and stroke. The figures display central risk estimates as solid lines, with shaded regions indicating the 95% confidence intervals. *p* values for both linear and nonlinear associations are reported. Nonlinearity was assessed using a likelihood ratio test comparing a fully adjusted spline model with a model containing only the linear term. All estimates were adjusted for confounders described in the footnote in Table 3.

During 99,751 person-years of follow-up (maximum: 18 years), a total of 597 incident CVD cases were documented. In multivariable models adjusted for demographic characteristics, medical history, lifestyle factors, and other cardiovascular risk factors, none of the three plant-based diet indices were significantly associated with overall CVD risk [PDI: HR for extreme quartiles (95% CI), 0.99 (0.80–1.24); *p*-trend = 0.779; hPDI: 1.06 (0.83–1.35); *p*-trend = 0.796; uPDI: 1.25 (0.97–1.62); *p*-trend = 0.070; Table 3]. When potential linear associations were evaluated using restricted cubic spline models, no linear associations were identified for all plant-based diet indices with CVD (Figure 2).

### 3.4. Exploratory and Sensitivity Analyses

When stratified by CVD subtypes, higher uPDI scores were significantly associated with an increased risk of CHD [HR (95% CI): 1.62 (1.12–2.33); *p*-trend = 0.008], whereas PDI and hPDI scores were not associated with CHD risk [PDI: HR for extreme quartiles (95% CI), 1.00 (0.74–1.34); *p*-trend = 0.962; hPDI: 0.99 (0.72–1.37); *p*-trend = 0.997, Table 3]. None of the three plant-based diet indices were significantly associated with the risk of stroke [PDI: HR for extreme quartiles (95% CI), 0.97 (0.71–1.33); *p*-trend = 0.705; hPDI: 1.13 (0.79–1.60); *p*-trend = 0.511; uPDI: 0.97 (0.66–1.42); *p*-trend = 0.964; Table 3]. When potential linear associations were evaluated using restricted cubic spline models, a marginally significant linear association was observed between uPDI and CHD (*p* for linearity = 0.05), whereas no linear associations were identified for PDI or hPDI with CVD subtypes (Figure 2). Exclusion of CVD events occurring in the first 2 years (*n* = 81) to minimize reverse causation due to preexisting subclinical disease partly attenuated the positive association of uPDI scores with CHD risk [HR for extreme quartiles (95% CI), 1.38 (0.94–2.04); *p*-trend = 0.094, Tables S3–S5]. Significant heterogeneity was not identified in the associations between all plant-based diet index scores and CVD according to age, sex, family history of CVD, physical activity, BMI, smoking status, or history of hypertension (Bonferroni-corrected *p*-interaction > 0.01 each). Similarly, the associations of PDI, hPDI, and uPDI with CVD subtypes were also generally consistent across subgroups (Figures S1–S3).

## 4. Discussion

In this large, community-based prospective cohort study among middle-aged and older Korean adults, none of the three plant-based diet indices (PDI, hPDI, uPDI) were significantly associated with overall risk of CVD. When stratified by CVD subtypes, the risk of CHD was positively associated with uPDI scores, but not with PDI and hPDI scores; and there was no association between PDI scores and stroke. To our knowledge, this is the first study to prospectively assess the associations of types of PDI scores and risk of CVD and its subtypes in a middle-aged and older Asian population.

The present study found no significant association of PDI, hPDI, or uPDI with the risk of CVD. These findings are partly consistent with prior evidence but differ from some prospective studies. A recent meta-analysis of 25 cohorts reported that higher hPDI scores were associated with lower risks of CVD, CHD, and stroke, whereas higher uPDI scores were linked to an increased risk of CVD but not to CHD or stroke [29]. Individual cohort studies, however, have reported mixed results. For example, in the ARIC study, higher PDI scores were inversely associated with CVD incidence, whereas hPDI and uPDI showed no association [13]. In contrast, the Jackson Heart Study observed no significant associations for any of the three indices [14], while the ATTICA study of middle-aged Greek adults found an inverse association only for hPDI [15]. These inconsistencies across studies may stem from differences in the definition of CVD (e.g., whether heart failure, arrhythmia, or angina were included), participant characteristics (Western vs. Asian populations, baseline cardiometabolic risk), and dietary assessment tools. Importantly, populations also differ in plant food consumption patterns; for example, vegetable intake averages 294.4 g/day in East Asia versus 123.3 g/day in North America [30], which may influence metabolic and genetic responses. In our cohort, the relatively high vegetable consumption and lower overall CVD incidence compared with Western cohorts may have attenuated associations [31]. CVD is a composite outcome encompassing heterogeneous subtypes, and the observed positive association of uPDI with CHD—but not with overall CVD or stroke—may reflect stronger contributions of refined grains, sugar-sweetened beverages, and salty foods to atherosclerotic processes in coronary arteries, whereas stroke risk may be influenced by additional factors not fully captured in our study. Such variations can influence the classification of foods into healthy or unhealthy plant-based categories and may partly explain the heterogeneity in PDIs–CVD associations. Future studies employing harmonized definitions of CVD and standardized dietary assessment tools are warranted to more clearly elucidate these associations.

When CVD subtypes were examined separately, we observed a positive association between uPDI scores and CHD risk, whereas no significant associations were found for PDI or hPDI. In contrast, none of the three indices were associated with stroke incidence. A recent meta-analysis reported directionally similar estimates for uPDI, with a relative risk of 1.12 (95% CI, 0.90–1.39) for CHD and 1.10 (0.90–1.34) for total stroke, with ischemic stroke (1.09; 0.96–1.25) showing a higher point estimate than hemorrhagic stroke (1.01; 0.82–1.20) [29]. Similar to our findings, the pooled analysis of the Nurses’ Health Study (NHS), NHSII, and Health Professionals Follow-up Study (HPFS) cohorts reported a positive association between uPDI and CHD [12], whereas no association was observed with total stroke [32]. In the UK Biobank study, uPDI was positively associated with ischemic stroke (HR 1.23; 95% CI, 1.04–1.46), whereas the association disappeared for hemorrhagic stroke (HR 1.06; 0.81–1.38) [16]. These patterns support the notion that unhealthy plant based diets—characterized by higher intakes of refined grains and sugar sweetened beverages—may more directly contribute to atherosclerotic processes in coronary and cerebral large arteries. Future studies are warranted to clarify the biological pathways through which uPDI may influence atherosclerotic disease development.

The development of CHD involves diverse biological mechanisms, including endothelial impairment, inflammation, insulin resistance, dyslipidemia, oxidative injury, and calcification of vessels [33,34]. Unhealthy dietary patterns—characterized by minimal whole grains, fruits, and vegetables and high levels of refined grains, sweetened drinks, and salty foods, as captured by the uPDI—are important contributors to this risk [9,35]. Prior mechanistic studies suggest that excessive sodium and sugar intake, along with inadequate fiber and micronutrients, may elevate oxidative stress and inflammation, damage mitochondria and vascular endothelium and ultimately promote CHD [36,37,38,39]. Further studies are needed to elucidate biological mechanisms linking uPDI to the development of CHD.

Our investigation has several strengths. The prospective design, with nearly 18 years of follow-up among a cohort of middle-aged and older adults, allowed for the accrual of a substantial number of cardiovascular events, thereby ensuring adequate statistical power. The community-based nature of the cohort enhances the external validity of our findings. Moreover, repeated assessments of diet and physical activity throughout the study period facilitated more accurate estimation of CVD risk factors and helped mitigate potential confounding.

Nonetheless, several limitations should be acknowledged. Although dietary intake was assessed using a validated 106-item FFQ, this instrument primarily captures broad food categories and lacks granularity in terms of preparation methods and food subtypes (e.g., low-sodium or sugar-added versions). Such limitations may introduce measurement error in nutrient intake estimations. Additionally, complex mixed dishes—such as pizza, hamburgers, and dumplings—were not deconstructed into their component ingredients, which may have led to misclassification of food groups. These limitations may have led to nondifferential misclassification of dietary exposures, potentially biasing associations toward the null. In addition, although dietary intake was assessed using a validated FFQ and nutrient intakes were estimated using the Korean Food Composition Table, only selected nutrients were available in the KoGES dataset. As a result, some nutrients of interest, such as vitamin D and magnesium, could not be examined in this study, which may limit the comprehensiveness of the dietary assessment. Future studies should employ more detailed dietary assessment instruments or incorporate biomarkers to improve dietary exposure classification. Furthermore, although our food group classification was based on existing evidence linking dietary components to chronic disease outcomes and metabolic risk, the health effects of some individual foods (e.g., potatoes, coffee) may differ depending on the disease in question, complicating efforts to categorize plant-based foods as uniformly healthful or unhealthful. Although dietary intake was assessed using FFQs at two time points, some degree of measurement error is inevitable, particularly over a long follow-up period. To minimize random error, we calculated cumulative averages to reduce random error, residual misclassification may still have biased the observed associations toward the null. Future studies with repeated dietary assessments at multiple time points and biomarker-based validation are warranted to more accurately capture long-term dietary exposures. Moreover, given the observational design, residual confounding from unmeasured or unknown variables may still be present. Variations in dietary habits and genetic backgrounds also suggest that these findings may not be generalizable beyond Korean populations, underscoring the need for replication in more diverse populations with different dietary patterns and genetic backgrounds to enhance the generalizability of these associations.

## 5. Conclusions

In conclusion, in this large, community-based prospective cohort of middle-aged and older Korean adults, none of the three plant-based diet indices (PDI, hPDI, uPDI) were significantly associated with overall CVD risk. In contrast, higher adherence to uPDI was associated with an increased risk of CHD, but not stroke. These findings underscore the importance of diet quality within plant-based patterns—distinguishing healthful from less-healthful options—when formulating strategies for CHD prevention. Replication in diverse populations is warranted to corroborate the associations of PDI, hPDI, and uPDI with CVD risk.

## Figures and Tables

**Table 1:** Baseline characteristics of participants across quartiles of plant-based diet scores.

	PDI		hPDI		uPDI	
	Quartile 1	Quartile 4	Quartile 1	Quartile 4	Quartile 1	Quartile 4
Sample size, *n* ^(1)^	2425	1922	1963	1913	2099	1633
Median score (range)	46 (31–48)	57 (55–70)	44 (29–46)	59 (56–74)	43 (30–46)	60 (57–75)
Women, *n* (%)	1129 (46.6)	1083 (56.4)	765 (39.1)	1215 (63.5)	1359 (64.8)	678 (41.5)
Age, years	50.3 ± 8.5	53.4 ± 9.0	50.3 ± 8.8	53.2 ± 8.7	49.9 ± 8.1	53.1 ± 9.0
Residential area, *n* (%)						
Rural, Ansung	1055 (43.5)	1045 (54.4)	772 (39.3)	1022 (53.4)	569 (27.1)	1161 (71.1)
Urban, Ansan	1370 (56.5)	877 (45.6)	1191 (60.7)	891 (46.6)	1530 (72.9)	742 (28.9)
Income (KRW/month), *n* (%)						
<1,000,000	646 (26.6)	772 (40.2)	527 (26.9)	716 (37.4)	422 (20.1)	735 (45.0)
1,000,000 to <2,000,000	737 (30.4)	545 (28.4)	583 (29.7)	584 (30.5)	587 (28.0)	505 (30.9)
2,000,000 to <3,000,000	522 (21.5)	315 (16.4)	423 (21.6)	313 (16.4)	514 (24.5)	222 (13.6)
≥3,000,000	520 (21.4)	290 (15.1)	430 (21.9)	300 (15.7)	576 (27.4)	171 (10.5)
Education level, *n* (%)						
≤Elementary school	571 (23.6)	784 (40.8)	504 (25.7)	714 (37.3)	401 (19.1)	679 (41.6)
Middle school	557 (23.0)	444 (23.1)	445 (22.7)	433 (22.6)	437 (20.8)	407 (24.9)
High school	848 (35.0)	511 (26.6)	655 (33.4)	548 (28.7)	843 (40.2)	398 (24.4)
≥College	449 (18.5)	183 (9.5)	359 (18.3)	218 (11.4)	418 (19.9)	149 (9.1)
Smoking (packs/year)	10.0 ± 15.6	9.0 ± 16.0	12.4 ± 16.6	7.2 ± 19.5	6.4 ± 13.5	11.8 ± 17.1
Alcohol intake (g/day)	11.7 ± 23.7	7.5 ± 20.1	13.0 ± 24.6	7.2 ± 19.5	8.4 ± 21.1	10.3 ± 21.9
Hypertension status, *n* (%)						
Hypertensive	693 (28.6)	654 (34.0)	556 (28.3)	671 (35.1)	586 (27.9)	553 (33.9)
Normotensive	1732 (71.4)	1268 (66.0)	1407 (71.7)	1242 (64.9)	1513 (72.1)	1080 (66.1)
Family history of CVD, *n* (%)						
Yes	379 (15.6)	272 (14.2)	317 (16.2)	272 (14.2)	343 (16.3)	223 (13.7)
No	2046 (84.4)	1650 (85.9)	1646 (83.9)	1641 (85.8)	1756 (83.7)	1410 (86.3)
Body mass index, kg/m^2^	24.4 ± 3.1	24.9 ± 3.2	24.4 ± 3.1	24.9 ± 3.2	24.8 ± 3.1	24.4 ± 3.2
Physical activity, METs/day	22.8 ± 14.1	26.0 ± 15.1	23.0 ± 14.3	24.6 ± 14.7	20.6 ± 12.1	28.1 ± 16.1
Total energy intake, kcal/day	1959.2 ± 519.0	1879.8 ± 468.9	1817.6 ± 518.8	2009.8 ± 471.8	1807.4 ± 482.4	2006.4 ± 527.4
Healthy plant foods, serv/d ^(2)^	10.4 ± 4.9	14.2 ± 5.5	9.3 ± 4.4	14.7 ± 5.6	14.2 ± 5.0	9.5 ± 4.9
Unhealthy plant foods, serv/d ^(3)^	7.2 ± 3.2	9.2 ± 3.6	9.5 ± 3.4	6.7 ± 3.2	6.4 ± 2.7	10.1 ± 3.7
Animal foods, serv/d ^(4)^	4.4 ± 2.4	3.1 ± 2.0	4.4 ± 2.3	3.1 ± 2.0	4.4 ± 2.2	2.9 ± 2.0

Values are presented as mean ± SD for continuous variables and as number (percentage) for categorical variables, unless otherwise indicated. ^(1)^ The number of participants per quartile varies because multiple individuals received identical scores. ^(2)^ Healthful plant foods include whole grains, fruits, vegetables, nuts, legumes, coffee, and green tea. ^(3)^ Less-healthful plant foods include refined grains, potatoes, sugar-sweetened beverages, sweets and desserts, and pickled vegetables or soybean paste. ^(4)^ Animal foods include meat, fish, eggs, dairy products, animal fats, and other animal-derived foods. Abbreviations: PDI, Plant-based Diet Index; hPDI, healthful Plant-based Diet Index; uPDI, unhealthful Plant-based Diet Index; MET, Metabolic Equivalent of Task.

**Table 2:** Nutrient intakes of study participants across quartiles of the plant-based diet indices.

	PDI		hPDI		uPDI	
	Quartile 1	Quartile 4	Quartile 1	Quartile 4	Quartile 1	Quartile 4
	(*n* = 2425)	(*n* = 1922)	(*n* = 1963)	(*n* = 1913)	(*n* = 2099)	(*n* = 1633)
Total energy intake, kcal/day	1959.2 ± 519.0	1886.9 ± 474.3	1817.6 ± 518.8	2009.8 ± 471.8	1807.4 ± 482.4	2006.4 ± 527.4
Carbohydrate, %E	68.8 ± 6.1	74.0 ± 5.0	69.5 ± 6.1	73.5 ± 5.4	68.8 ± 5.2	74.4 ± 5.7
Protein, %E	13.8 ± 2.1	12.9 ± 1.8	13.6 ± 2.1	13.0 ± 1.9	14.5 ± 1.8	12.0 ± 1.7
Fat, %E	16.1 ± 4.8	12.3 ± 4.0	15.7 ± 4.8	12.4 ± 4.2	16.0 ± 4.1	12.2 ± 4.7
Vitamin A, μg RE	241.7 ± 113.1	296.8 ± 138.1	270.2 ± 123.9	261.6 ± 138.2	295.5 ± 125.3	239.9 ± 122.0
Vitamin E, mg	4.4 ± 1.2	4.9 ± 1.3	4.61 ± 1.21	4.63 ± 1.35	5.2 ± 1.2	4.1 ± 1.2
Vitamin B1, mg	0.61 ± 0.12	0.61 ± 0.10	0.61 ± 0.11	0.61 ± 0.11	0.64 ± 0.10	0.57 ± 0.11
Vitamin B2, mg	0.52 ± 0.12	0.49 ± 0.12	0.52 ± 0.12	0.48 ± 0.13	0.58 ± 0.12	0.43 ± 0.10
Niacin, mg	7.9 ± 1.5	8.0 ± 1.3	8.1 ± 1.4	7.7 ± 1.3	8.6 ± 1.3	7.1 ± 1.2
Vitamin B6, mg	0.87 ± 0.15	0.95 ± 0.16	0.90 ± 0.15	0.90 ± 0.17	1.0 ± 0.1	0.8 ± 0.2
Folate, μg	110.0 ± 33.0	141.6 ± 39.7	119.2 ± 37.1	130.0 ± 41.0	135.9 ± 36.2	112.3 ± 37.5
Vitamin C, mg	52.8 ± 27.7	73.6 ± 29.4	56.4 ± 23.1	67.5 ± 34.1	70.1 ± 28.6	54.4 ± 27.1
Calcium, mg	239.9 ± 83.8	247.4 ± 79.9	245.8 ± 81.8	235.5 ± 84.6	284.0 ± 85.3	197.6 ± 68.7
Phosphorus, mg	515.9 ± 75.4	517.0 ± 72.4	511.8 ± 73.8	518.0 ± 75.7	566.5 ± 71.4	457.3 ± 59.0
Sodium, mg	1421.5 ± 515.8	1829.8 ± 633.9	1758.1 ± 632.7	1479.2 ± 581.9	1567.9 ± 559.2	1662.3 ± 632.3
Potassium, mg	1189.7 ± 289.8	1418.5 ± 310.6	1257.1 ± 293.3	1316.3 ± 339.7	1459.6 ± 303.3	1115.1 ± 272.0
Iron, mg	5.2 ± 1.1	5.9 ± 1.2	5.2 ± 1.2	5.8 ± 1.2	6.2 ± 1.1	4.7 ± 1.0
Zinc, μg	4.6 ± 1.0	4.3 ± 0.7	4.43 ± 0.94	4.37 ± 0.78	4.8 ± 1.1	4.0 ± 0.8
Fiber, g	3.0 ± 0.8	4.1 ± 1.0	3.2 ± 0.9	3.8 ± 1.1	3.7 ± 1.0	3.3 ± 1.0
Cholesterol, mg	102.6 ± 44.8	66.0 ± 37.2	100.1 ± 45.1	67.8 ± 39.3	107.4 ± 41.7	60.1 ± 37.2

Macronutrients were reported as percent of total energy intake, and micronutrient levels were calculated relative to 1000 kcal of energy. Abbreviations: PDI, Plant-based Diet Index; hPDI, healthful Plant-based Diet Index; uPDI, unhealthful Plant-based Diet Index.

**Table 3:** Risk of incident CVD and its subtypes associated with plant-based diet indices among 7820 adults in the Korean Genome and Epidemiology Study (KoGES)_Ansan and Ansung study.

	Quartiles of Plant-Based Diet Index Scores				*p*-Trend ^(1)^
	Quartile 1	Quartile 2	Quartile 3	Quartile 4	
Cardiovascular disease (*n* = 597)					
Overall plant-based diet index					
Median score (range)	46 (31–48)	50 (49–51)	53 (52–54)	57 (55–70)	
Cases/total	174/2425	139/1826	119/1647	165/1922	
Person-years	30,521	23,188	21,432	24,608	
Age, sex adjusted	Reference	0.98 (0.79–1.23)	0.87 (0.69–1.10)	1.01 (0.81–1.25)	0.846
Multivariable adjusted *	Reference	0.98 (0.78–1.22)	0.86 (0.68–1.09)	0.99 (0.80–1.24)	0.779
Healthful plant-based diet index					
Median score	44 (29–46)	49 (47–51)	53 (52–55)	59 (56–74)	
Cases/total	135/1963	179/2258	129/1686	154/1913	
Person-years	23,991	29,071	21,948	24,741	
Age, sex adjusted	Reference	1.05 (0.84–1.31)	0.98 (0.76–1.24)	1.02 (0.81–1.29)	0.986
Multivariable adjusted *	Reference	1.07 (0.86–1.35)	1.00 (0.78–1.28)	1.06 (0.83–1.35)	0.796
Unhealthful plant-based diet index					
Median score	43 (30–46)	49 (47–51)	54 (52–56)	60 (57–75)	
Cases/total	135/2099	150/2069	163/2019	149/1633	
Person-years	27,579	26,709	25,350	20,113	
Age, sex adjusted	Reference	0.99 (0.78–1.25)	1.04 (0.82–1.31)	1.18 (0.93–1.50)	0.151
Multivariable adjusted *	Reference	0.99 (0.78–1.25)	1.07 (0.84–1.36)	1.25 (0.97–1.62)	0.07
Coronary heart disease (*n* = 314)					
Overall plant-based diet index					
Median score (range)	46 (31–48)	50 (49–51)	53 (52–54)	57 (55–70)	
Cases/total	94/2425	66/1834	66/1636	88/1925	
Person-years	30,901	23,738	21,433	25,036	
Age, sex adjusted	Reference	0.86 (0.62–1.17)	0.91 (0.66–1.25)	1.00 (0.74–1.34)	0.962
Multivariable adjusted *	Reference	0.85 (0.62–1.17)	0.90 (0.66–1.24)	0.97 (0.72–1.30)	0.88
Healthful plant-based diet index					
Median score	44 (29–46)	49 (47–51)	53 (52–55)	59 (56–74)	
Cases/total	76/1962	88/2257	66/1683	84/1918	
Person-years	24,264	29,489	22,207	25,147	
Age, sex adjusted	Reference	0.92 (0.68–1.26)	0.90 (0.65–1.26)	1.00 (0.73–1.37)	0.963
Multivariable adjusted *	Reference	0.92 (0.67–1.25)	0.90 (0.65–1.26)	0.99 (0.72–1.37)	0.997
Unhealthful plant-based diet index					
Median score	43 (30–46)	49 (47–51)	54 (52–56)	60 (57–75)	
Cases/total	60/2099	79/2074	88/2011	87/1636	
Person-years	27,904	27,156	25,611	20,436	
Age, sex adjusted	Reference	1.18 (0.84–1.65)	1.28 (0.92–1.79)	1.56 (1.12–2.19)	0.008
Multivariable adjusted *	Reference	1.19 (0.84–1.67)	1.31 (0.93–1.85)	1.62 (1.12–2.33)	0.008
Stroke (*n* = 287)					
Overall plant-based diet index					
Median score (range)	46 (32–48)	50 (49–51)	53 (52–54)	57 (55–70)	
Cases/total	85/2426	69/1836	57/1637	76/1921	
Person-years	31,034	23,812	21,689	25,163	
Age, sex adjusted	Reference	0.99 (0.72–1.36)	0.86 (0.61–1.20)	0.95 (0.69–1.30)	0.611
Multivariable adjusted *	Reference	0.98 (0.71–1.36)	0.85 (0.61–1.20)	0.97 (0.71–1.33)	0.705
Healthful plant-based diet index					
Median score	44 (29–46)	49 (47–51)	53 (52–55)	59 (56–74)	
Cases/total	65/1971	85/2251	64/1684	73/1914	
Person-years	24,531	29,583	22,352	25,232	
Age, sex adjusted	Reference	1.05 (0.76–1.46)	1.03 (0.73–1.45)	1.03 (0.73–1.45)	0.922
Multivariable adjusted *	Reference	1.11 (0.80–1.53)	1.09 (0.77–1.55)	1.13 (0.79–1.60)	0.511
Unhealthful plant-based diet index					
Median score	43 (30–46)	49 (47–51)	54 (52–56)	60 (57–75)	
Cases/total	71/2094	75/2077	82/2022	59/1627	
Person-years	27,881	27,325	25,837	20,654	
Age, sex adjusted	Reference	1.05 (0.76–1.46)	1.03 (0.73–1.45)	1.03 (0.73–1.45)	0.427
Multivariable adjusted *	Reference	0.93 (0.66–1.29)	1.03 (0.74–1.45)	0.97 (0.66–1.42)	0.964

^(1)^ For trend analysis, the median value of each quartile was assigned to participants and modeled as a continuous variable. * The fully adjusted model included the following covariates: age (years, continuous), sex (male/female), residential area (urban/rural), household income (<1,000,000; 1,000,000–<2,000,000; 2,000,000–<3,000,000; ≥3,000,000), education level (≤elementary, middle school, high school, ≥associate’s degree), smoking (packs per year, continuous), alcohol intake (grams per day, continuous), family history of CVD (yes/no), history of hypertension (yes/no), body mass index (kg/m^2^, continuous), physical activity (METs per day, continuous), and total energy intake (kcal/day, continuous).

## Data Availability

The data are publicly available at https://coda.nih.go.kr/frt/index.do (accessed on 5 October 2022).

## Supplementary Materials

The following supporting information can be downloaded at https://www.mdpi.com/article/10.3390/nu17172805/s1, Table S1. STROBE Statement—Checklist of items that should be included in reports of cohort studies; Table S2. Scoring system and classification based on food group composition in the Korean Genome and Epidemiology Study; Table S3. Sensitivity analyses for the associations between plant-based diet indices and risk of CVD among 7739 men and women in the Korean Genome and Epidemiology Study (KoGES)_Ansan and Ansung study excluding events within the first 2 years of follow-up; Table S4. Sensitivity analyses for the associations between plant-based diet indices and risk of coronary heart disease among 7775 men and women in the Korean Genome and Epidemiology Study (KoGES)_Ansan and Ansung study excluding events within the first 2 years of follow-up; Table S5. Sensitivity analyses for the associations between plant-based diet indices and risk of stroke among 7788 men and women in the Korean Genome and Epidemiology Study (KoGES)_Ansan and Ansung study excluding events within the first 2 years of follow-up; Figure S1. Plant-based diet indices and the risk of cardiovascular disease in the Korean Genome and Epidemiology Study (KoGES)_Ansan and Ansung study: analysis of potential interaction by age, sex, family history of CVD, physical activity, BMI, smoking status, and history of hypertension; Figure S2. Plant-based diet indices and the risk of coronary heart disease in the Korean Genome and Epidemiology Study (KoGES)_Ansan and Ansung study: analysis of potential interaction by age, sex, family history of CVD, physical activity, BMI, smoking status, and history of hypertension; Figure S3. Plant-based diet indices and the risk of stroke in the Korean Genome and Epidemiology Study (KoGES)_Ansan and Ansung study: analysis of potential interaction by age, sex, family history of CVD, physical activity, BMI, smoking status, and history of hypertension.

## Abbreviations

The following abbreviations are used in this manuscript:

PDI	Overall Plant-based Diet Index
hPDI	Healthful Plant-based Diet Index
uPDI	Unhealthful Plant-based Diet Index
