# Supplementary material for: Plant-Based Dietary Patterns and the Risk of Cardiovascular Disease in Middle-Aged Korean Adults: A Community-Based Prospective Cohort Study
Source: Nutrients. 2025 Aug 28;17(17):2805. doi: 10.3390/nu17172805 (PMC12430265; doi:10.3390/nu17172805)
Supplement: Supplementary file 1 [file nutrients-17-02805-s001.zip › nutrients-3827414-supplementary - UPDATE.pdf]

Supplementary Materials

Table S1. STROBE Statement—Checklist of items that should be included in reports of ***cohort studies***

	Item No	Recommendation	Page
**Title and abstract**	1	(*a*) Indicate the study's design with a commonly used term in the title or the abstract	1
		(*b*) Provide in the abstract an informative and balanced summary of what was done and what was found	1
**Introduction**			
Background/rationale	2	Explain the scientific background and rationale for the investigation being reported	1-2
Objectives	3	State specific objectives, including any prespecified hypotheses	2
**Methods**			
Study design	4	Present key elements of study design early in the paper	2
Setting	5	Describe the setting, locations, and relevant dates, including periods of recruitment, exposure, follow-up, and data collection	2
Participants	6	(*a*) Give the eligibility criteria, and the sources and methods of selection of participants. Describe methods of follow-up	2
		(*b*) For matched studies, give matching criteria and number of exposed and unexposed	
Variables	7	Clearly define all outcomes, exposures, predictors, potential confounders, and effect modifiers. Give diagnostic criteria, if applicable	4-6
Data sources/ measurement	8*	For each variable of interest, give sources of data and details of methods of assessment (measurement). Describe comparability of assessment methods if there is more than one group	3-4
Bias	9	Describe any efforts to address potential sources of bias	4-5
Study size	10	Explain how the study size was	3

		arrived at	
Quantitative variables	11	Explain how quantitative variables were handled in the analyses. If applicable, describe which groupings were chosen and why	4-5
Statistical methods	12	(*a*) Describe all statistical methods, including those used to control for confounding	4-5
		(*b*) Describe any methods used to examine subgroups and interactions	4-5
		(*c*) Explain how missing data were addressed	2
		(*d*) If applicable, explain how loss to follow-up was addressed	4
		(*e*) Describe any sensitivity analyses	5
**Results**			
Participants	13*	(a) Report numbers of individuals at each stage of study—eg numbers potentially eligible, examined for eligibility, confirmed eligible, included in the study, completing follow-up, and analysed	2
		(b) Give reasons for non-participation at each stage	
		(c) Consider use of a flow diagram	Figure 1
Descriptive data	14*	(a) Give characteristics of study participants (eg demographic, clinical, social) and information on exposures and potential confounders	Table 1
		(b) Indicate number of participants with missing data for each variable of interest	2
		(c) Summarise follow-up time (eg, average and total amount)	4
Outcome data	15*	Report numbers of outcome events or summary measures over time	Table 3
Main results	16	(*a*) Give unadjusted estimates and, if applicable, confounder-adjusted estimates and their precision (eg, 95% confidence interval). Make clear which confounders were adjusted for and why they were included	Table 3

		(*b*) Report category boundaries when continuous variables were categorized	Table 3
		(*c*) If relevant, consider translating estimates of relative risk into absolute risk for a meaningful time period	
Other analyses	17	Report other analyses done—eg analyses of subgroups and interactions, and sensitivity analyses	Tables S3-S5<br>Figures S1-S3
**Discussion**			
Key results	18	Summarise key results with reference to study objectives	11
Limitations	19	Discuss limitations of the study, taking into account sources of potential bias or imprecision. Discuss both direction and magnitude of any potential bias	12
Interpretation	20	Give a cautious overall interpretation of results considering objectives, limitations, multiplicity of analyses, results from similar studies, and other relevant evidence	11-12
Generalisability	21	Discuss the generalisability (external validity) of the study results	12
**Other information**			
Funding	22	Give the source of funding and the role of the funders for the present study and, if applicable, for the original study on which the present article is based	12

*Give information separately for exposed and unexposed groups.

Table S2. Scoring system and classification based on food group composition in the Korean Genome and Epidemiology Study

Food Groups	Food subgroups	Items in the food frequency questionnaire	PDI	hPDI	uPDI
Healthy plant foods	Whole grains	Mixed grains, barley, grain with beans	+	+	-
	Fruits	Strawberry, watermelon, banana, peach/plum, oriental melon/melon, persimmon/dried persimmon, pear/pear juice, tangerine, orange/orange juice, apple/apple juice, grape/grape juice			
	Vegetables	Sweet potatoes, radish, napa cabbage/napa cabbage soup, spinach, lettuce, perilla leaves, sesame leaves/vegetable salad, other green vegetable, codonopsis pilosula /bellflower root, bean sprouts/mung-bean sprouts, bracken/sweet potato stem, oyster mushroom, other mushrooms, green pepper leaf/chamnamul, cucumber, carrot/carrot juice crown daisy/chive/watercress, onion, green peppers, zucchini, pumpkin/kabocha squash, laver, kelp/seaweed, tomato/tomato juice			
	Nuts	Peanuts/almonds/pine nuts			
	Legumes	Beans/beans cooked in soy sauce, tofu, bean curd, soybean milk			
	Green tea and coffee	Coffee, green tea			
Less healthy plant foods	Refined grains	White rice, instant noodles, other noodles (udon noodles), black bean sauce noodles, cold noodles, rice cake/rice cake soup, other rice cakes, cereals, white breads, other breads, grain powder, starch jelly, stir-fried noodles and vegetables	+	-	+
	Potatoes	Potatoes			
	Sugar-sweetened beverages	Soft drink, other beverages (sweetened rice tea, citron tea)			
	Sweets and desserts	Sweet red bean bread, cake/chocolate pie, cookies/crackers/snacks, candies/chocolates, sugars (added to tea or coffee)			
	Pickled vegetables and soybean paste	Bean paste, bean paste/bean paste soup, kimchi (Korean cabbage, radish), watery radish kimchi, other kimchi, pickled vegetable (preserved in soy sauce or salt), radish kimchi (preserved in soy sauce or salt)			
Animal foods	Animal fat	Butter, cream (added to tea or coffee)	-	-	-
	Dairy	Milk, yogurt/yoplait, ice cream, cheese			
	Eggs	Eggs/quail eggs			
	Fish	Sashimi, belt fish, mackerel/pacific saury, eel, yellow croaker/sea bream/sole, alaska pollack/frozen pollack/dried pollack, squid/dried squid/octopus, anchovy/stir-fried anchovy, canned tuna, salted shrimp/salted fish, clam/sea snail, oyster, crab/marinated crab, shrimp, fishcake			
	Meat	Pork belly, grilled pork/stir-fried pork/pork bulgogi/Korean meatball, steamed pork, processed meat (ham, sausage), organ meat/korean sausage, steak/grilled beef, dog meat, beef soup, beef stew, chicken (fried, stew, braised spicy chicken),			
	Other animal foods	Dumplings/dumpling soup, pizza/hamburger			

Table S3. Sensitivity analyses for the associations between plant-based diet indices and risk of CVD among 7,739 men and women in the Korean Genome and Epidemiology Study (KoGES)_Ansan and Ansung study excluding events within the first 2 years of follow-up

	Quartiles of plant-based diet index scores				*P*-trend[1)]
	Quartile 1	Quartile 2	Quartile 3	Quartile 4	
Overall plant-based diet index					
Median score (range)	46 (31-48)	50 (49-51)	53 (52-54)	57 (55-70)	
Cases/total	146/2,397	118/1,805	108/1,636	144/1,901	
Person-years	30,470	23,149	21,412	24,569	
Age, sex adjusted	Reference	1.01 (0.79-1.29)	0.95 (0.74-1.22)	1.06 (0.84-1.34)	0.701
Multivariable adjusted*	Reference	0.99 (0.77-1.26)	0.93 (0.72-1.20)	1.05 (0.83-1.33)	0.773
Healthful plant-based diet index					
Median score	44 (29-46)	49 (47-51)	53 (52-55)	59 (56-74)	
Cases/total	118/1,946	155/2,234	109/1,666	134/1,893	
Person-years	23,960	29,026	21,911	24,703	
Age, sex adjusted	Reference	1.05 (0.82-1.33)	0.95 (0.73-1.24)	1.03 (0.80-1.33)	0.964
Multivariable adjusted*	Reference	1.07 (0.84-1.36)	0.98 (0.75-1.28)	1.10 (0.85-1.42)	0.611
Unhealthful plant-based diet index					
Median score	43 (30-46)	49 (47-51)	54 (52-56)	60 (57-75)	
Cases/total	126/2,090	130/2,049	138/1,994	122/1,606	
Person-years	27,562	26,672	25,302	20,063	
Age, sex adjusted	Reference	0.92 (0.72-1.18)	0.94 (0.73-1.20)	1.03 (0.80-1.33)	0.787
Multivariable adjusted*	Reference	0.92 (0.71-1.18)	0.97 (0.75-1.25)	1.09 (0.83-1.44)	0.491

1) To assess linear trends, quartiles were assessed as linear variables after assigning participants the median value in each quintile. * In all analyses, the multivariable-adjusted model was adjusted for age(year, continuous), sex(men/women), residence area(rural/urban), income(<1,000,000/1,000,000 to <2,000,000/2,000,000 to <3,000,000/≥3,000,000), education(≤elementary school graduation/middle school graduation/high school graduation/≥associate's degree graduation), smoking(packs/year, continuous), alcohol intake(gram/day, continuous), family history of CVD(yes/no), history of hypertension(yes/no), body mass index(kg/m2, continuous), physical activity(MET/day, continuous), and total energy intake(kcal/day, continuous).

Table S4. Sensitivity analyses for the associations between plant-based diet indices and risk of coronary heart disease among 7,775 men and women in the Korean Genome and Epidemiology Study (KoGES)_Ansan and Ansung study excluding events within the first 2 years of follow-up

	Quartiles of plant-based diet index scores				*P*-trend[1)]
	Quartile 1	Quartile 2	Quartile 3	Quartile 4	
Overall plant-based diet index					
Median score (range)	46 (31-48)	50 (49-51)	53 (52-54)	57 (55-70)	
Cases/total	78/2,409	57/1,825	60/1,630	74/1,911	
Person-years	30870	23721	21422	25010	
Age, sex adjusted	Reference	0.90 (0.64-1.27)	1.01 (0.72-1.41)	1.03 (0.74-1.42)	0.777
Multivariable adjusted*	Reference	0.89 (0.63-1.26)	0.99 (0.71-1.40)	1.00 (0.72-1.38)	0.905
Healthful plant-based diet index					
Median score	44 (29-46)	49 (47-51)	53 (52-55)	59 (56-74)	
Cases/total	67/1,953	76/2,245	52/1,669	74/1,908	
Person-years	24248	29467	22181	25128	
Age, sex adjusted	Reference	0.91 (0.66-1.27)	0.82 (0.57-1.18)	1.02 (0.73-1.43)	0.935
Multivariable adjusted*	Reference	0.91 (0.65-1.26)	0.82 (0.57-1.18)	1.04 (0.73-1.47)	0.855
Unhealthful plant-based diet index					
Median score	43 (30-46)	49 (47-51)	54 (52-56)	60 (57-75)	
Cases/total	56/2,095	68/2,063	74/1,997	71/1,620	
Person-years	27,897	27,135	25,584	20,408	
Age, sex adjusted	Reference	1.08 (0.76-1.54)	1.14 (0.80-1.62)	1.35 (0.94-1.93)	0.097
Multivariable adjusted*	Reference	1.09 (0.76-1.56)	1.16 (0.80-1.68)	1.38 (0.94-2.04)	0.094

1) To assess linear trends, quartiles were assessed as linear variables after assigning participants the median value in each quintile. * In all analyses, the multivariable-adjusted model was adjusted for age(year, continuous), sex(men/women), residence area(rural/urban), income(<1,000,000/1,000,000 to <2,000,000/2,000,000 to <3,000,000/≥3,000,000), education(≤elementary school graduation/middle school graduation/high school graduation/≥associate's degree graduation), smoking(packs/year, continuous), alcohol intake(gram/day, continuous), family history of CVD(yes/no), history of hypertension(yes/no), body mass index(kg/m2, continuous), physical activity(MET/day, continuous), and total energy intake(kcal/day, continuous).

Table S5. Sensitivity analyses for the associations between plant-based diet indices and risk of stroke among 7,788 men and women in the Korean Genome and Epidemiology Study (KoGES)_Ansan and Ansung study excluding events within the first 2 years of follow-up

	Quartiles of plant-based diet index scores				*P*-trend[1)]
	Quartile 1	Quartile 2	Quartile 3	Quartile 4	
Overall plant-based diet index					
Median score (range)	46 (32-48)	50 (49-51)	53 (52-54)	57 (55-70)	
Cases/total	75/2,416	58/1,825	53/1,633	69/1,914	
Person-years	31,016	23,791	21,681	25,150	
Age, sex adjusted	Reference	0.95 (0.67-1.34)	0.91 (0.64-1.30)	0.99 (0.71-1.38)	0.910
Multivariable adjusted*	Reference	0.93 (0.66-1.31)	0.89 (0.62-1.28)	1.01 (0.72-1.41)	0.991
Healthful plant-based diet index					
Median score	44 (29-46)	49 (47-51)	53 (52-55)	59 (56-74)	
Cases/total	59/1,965	76/2,242	56/1,676	64/1,905	
Person-years	24,520	29,566	22,337	25,214	
Age, sex adjusted	Reference	1.04 (0.74-1.47)	1.00 (0.69-1.44)	1.01 (0.70-1.44)	0.958
Multivariable adjusted*	Reference	1.09 (0.78-1.54)	1.06 (0.73-1.54)	1.11 (0.77-1.61)	0.617
Unhealthful plant-based diet index					
Median score	43 (30-46)	49 (47-51)	54 (52-56)	60 (57-75)	
Cases/total	66/2,089	67/2,069	71/2,011	51/1,619	
Person-years	27,872	27,311	25,816	20,638	
Age, sex adjusted	Reference	0.89 (0.63-1.25)	0.90 (0.64-1.27)	0.79 (0.54-1.14)	0.243
Multivariable adjusted*	Reference	0.90 (0.63-1.27)	0.97 (0.68-1.39)	0.92 (0.61-1.37)	0.779

1) To assess linear trends, quartiles were assessed as linear variables after assigning participants the median value in each quintile. * In all analyses, the multivariable-adjusted model was age(year, continuous), sex(men/women), residence area(rural/urban), income(<1,000,000/1,000,000 to <2,000,000/2,000,000 to <3,000,000/≥3,000,000), education(≤elementary school graduation/middle school graduation/high school graduation/≥associate's degree graduation), smoking(packs/year, continuous), alcohol intake(gram/day, continuous), family history of CVD(yes/no), history of hypertension(yes/no), body mass index(kg/m2, continuous), physical activity(MET/day, continuous), and total energy intake(kcal/day, continuous).

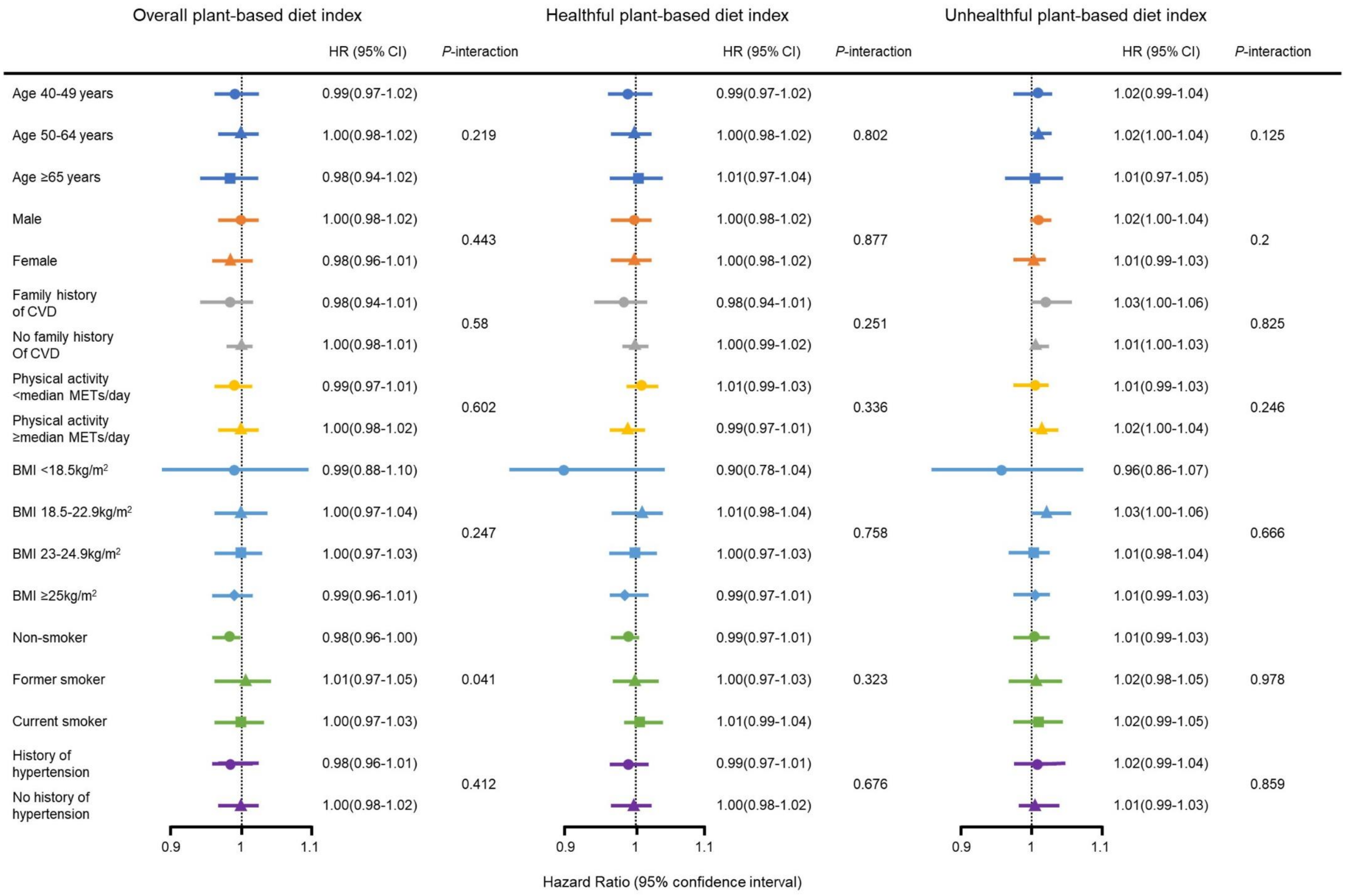

Figure S1. Plant-based diet indices and the risk of cardiovascular disease in the Korean Genome and Epidemiology Study (KoGES)_Ansan and Ansung study: analysis of potential interaction by age, sex, family history of CVD, physical activity, BMI, smoking status, and history of hypertension with respective stratified analyses with Bonferroni correction (significance<0.007).

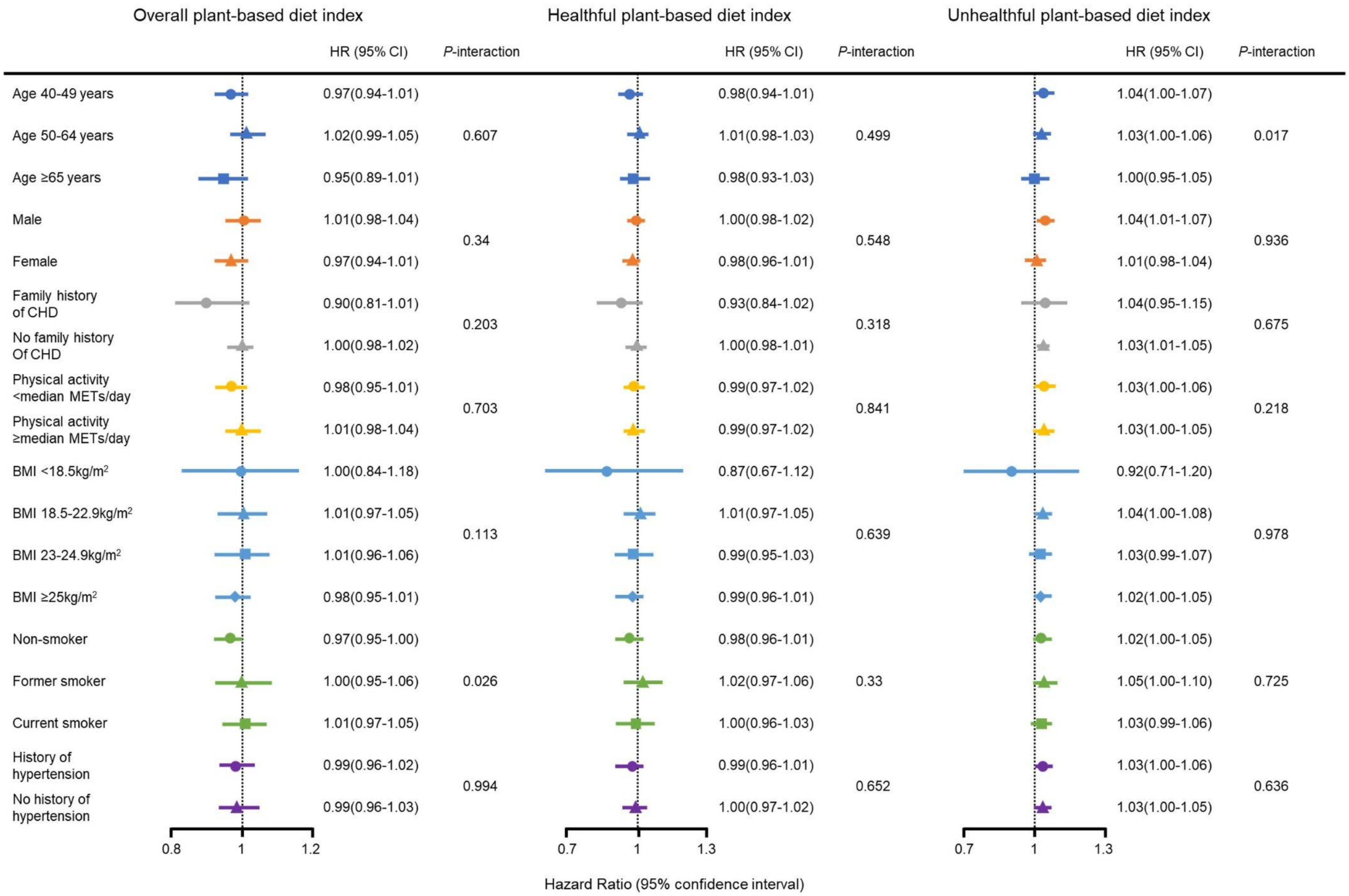

Figure S2. Plant-based diet indices and the risk of coronary heart disease in the Korean Genome and Epidemiology Study (KoGES)_Ansan and Ansung study: analysis of potential interaction by age, sex, family history of CVD, physical activity, BMI, smoking status, and history of hypertension with respective stratified analyses with Bonferroni correction (significance<0.007).

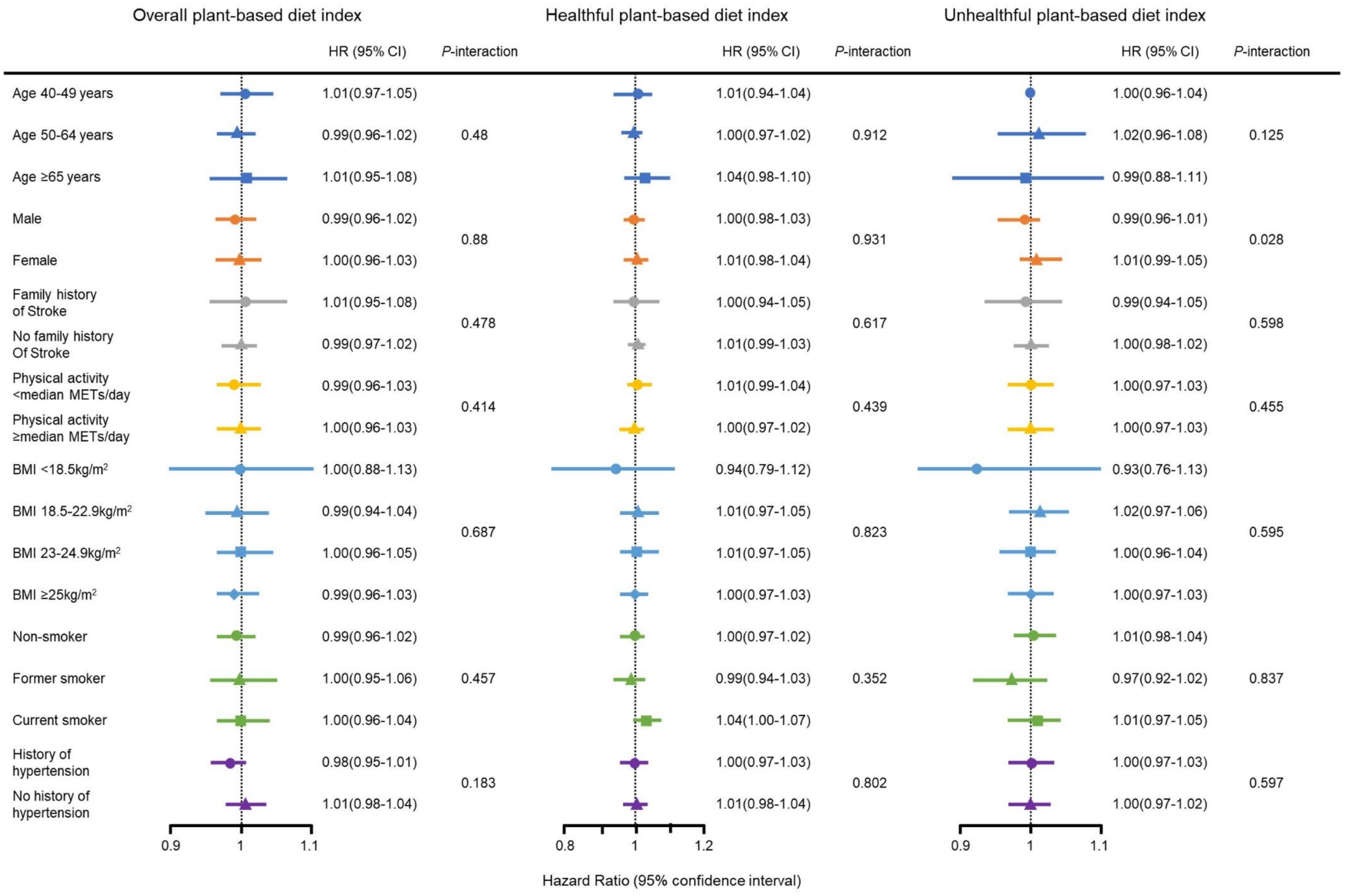

Figure S3. Plant-based diet indices and the risk of stroke in the Korean Genome and Epidemiology Study (KoGES)_Ansan and Ansung study: analysis of potential interaction by age, sex, family history of CVD, physical activity, BMI, smoking status, and history of hypertension with respective stratified analyses with Bonferroni correction (significance<0.007).
